# Relationship between Extension or Texture Features of Late Gadolinium Enhancement and Ventricular Tachyarrhythmias in Hypertrophic Cardiomyopathy

**DOI:** 10.1155/2018/4092469

**Published:** 2018-09-09

**Authors:** Yasuo Amano, Yasuyuki Suzuki, Fumi Yanagisawa, Yuko Omori, Naoya Matsumoto

**Affiliations:** ^1^Department of Radiology, Nihon University Hospital, 1-6 Kanda-Surugadai, Chiyoda-ku, Tokyo 101-8309, Japan; ^2^Department of Cardiology, Nihon University Hospital, Tokyo, Japan

## Abstract

**Purpose:**

To evaluate the relationship between extension or texture features of late gadolinium enhancement (LGE) and ventricular tachyarrhythmias in hypertrophic cardiomyopathy (HCM).

**Materials and Methods:**

Twenty-three patients with HCM were enrolled in this IRB-approved study. The extension of LGE was determined based on the American Heart Association segments model. Texture analysis was performed for 43 myocardial LGE using an open-access software (MaZda, Technical University of Lodz, Institute of Electronics, Poland). The relationship between the extension or texture features of LGE and ventricular tachyarrhythmias was evaluated using unpaired test and receiver-operating characteristic (ROC) analysis.

**Results:**

Six of 23 patients had a history of ventricular tachyarrhythmias, and 16 patients had LGE. All of the 6 patients with the arrhythmias had more than 4 LGE segments and more LGE segments than those without (*p* < 0.01). Among 4 texture features, entropy LL was the only discriminator between the 2 patient groups (*p* < 0.01; threshold, 19624; area under the curve [AUC], 0.72). An ROC analysis gave the number of segments showing LGE a better result (AUC, 0.96) for identification of HCM patients with ventricular tachyarrhythmias than the entropy LL of LGE.

**Conclusion:**

Patients with HCM and a history of ventricular tachyarrhythmias had a wider extension of LGE, and their entropy LL of LGE was significantly lower than those without. The extension of LGE and texture analysis may provide information about LGE related to ventricular tachyarrhythmias in HCM.

## 1. Introduction

Hypertrophic cardiomyopathy (HCM) is a relatively frequent, autosomal dominant transmitted myocardial disease [1]. Ventricular tachyarrhythmia is one of the most serious complications of HCM and a risk factor for sudden cardiac death, which can be prevented using the appropriate installation of an implantable cardioverter defibrillator [1–4]. In addition to some clinical risk factors, including a family history of sudden cardiac death and massive myocardial hypertrophy, late gadolinium enhancement (LGE) magnetic resonance (MR) imaging is useful in identifying myocardial fibrosis related to the ventricular tachyarrhythmia. Several studies have demonstrated that the presence or extension of myocardial LGE is related to ventricular tachyarrhythmia in HCM [5–8]. By contrast, intermediate-signal-intensity LGE has been reported to be related to ventricular tachyarrhythmia in patients with HCM [9]. Anyhow, these previous studies have defined LGE according to the visual inspection or threshold of standard deviation (SD) above the signal intensity of the remote normal-appearing myocardium, and thus other quantitative postprocessing may be required to establish the link between LGE and the arrhythmia.

Texture analysis is a quantitative analysis of medical images [10–18]. The texture of images refers to the histogram, distribution, gradient, correlation of neighbor pixels, and randomness of signal intensity on MRI [10, 12]. MRI of a wide variety of diseases, such as breast carcinoma, neurological diseases, or liver fibrosis, is assessed using texture analysis [10–18]. In the field of cardiac MRI, Kotu et al. [16] have assessed texture features to identify myocardial scarring associated with myocardial infarction. Baessler et al. [17] have shown that the texture analysis using an open-access software depicts subacute and chronic myocardial infarction on non-contrast-enhanced cine MRI. So far, to our knowledge, texture analysis has not been used to define myocardial LGE related to ventricular tachyarrhythmia in patients with HCM. The purpose of this study was to evaluate the texture features of LGE that are related to a history of ventricular tachyarrhythmias in patients with HCM.

## 2. Method and Materials

### 2.1. Study Design and Patients

This retrospective, cross-sectional, observational study was approved by our institutional review board, and informed consent was given by all the patients. Twenty-three consecutive patients HCM were referred to cardiac MR imaging examinations between August 2011 and April 2017 and enrolled in this study. They were 19 men and 4 women, ranging in age from 38 to 90 years (mean, 59.2 years). The phenotypes of HCM were basal asymmetrical septal hypertrophy (ASH; n = 10), midventricular obstruction (MVO) combined with apical HCM (APH; n = 5), APH (n = 4), dilated phase HCM (n = 3), and basal ASH combined with MVO (n = 1). One patient had an apparent family history of HCM. In all 23 patients, cine MR imaging at end-diastole showed a maximum myocardial thickness of > 15 mm in the absence of other myocardial diseases leading to myocardial hypertrophy.

### 2.2. MR Imaging

Cardiac MR imaging examinations were performed using a 3.0 T scanner (n = 12) or a 1.5 T scanner (n = 11) (Philips Medical Systems, Best, The Netherlands). Breath-hold 2-dimensional (2D) cine steady-state free precession imaging was performed on the short-axis, and in the 2-, 3- and 4-chamber view planes. A single-breath-hold 3D LGE MR imaging was performed on the same view planes as cine imaging approximately 10 min after an injection of 0.10 mmol/kg gadolinium-based contrast agents. The typical imaging parameters of the 3D LGE imaging included repetition time 3.7-4.2 ms, echo time 1.12-1.8 ms, flip angle 15°, in plane resolution 1.40-1.59 × 2.05-3.17 mm^2^, and slice thickness 10 mm followed by 5 mm interpolation. The inversion time nullifying the myocardium effectively (i.e., normal-appearing myocardium showing delayed signal recovery compared with the blood) was determined in each patient based on T1-weighted scout (Look-Locker) MR imaging.

### 2.3. LGE Imaging Visual Analysis and Texture Analysis

**Figure 1:**
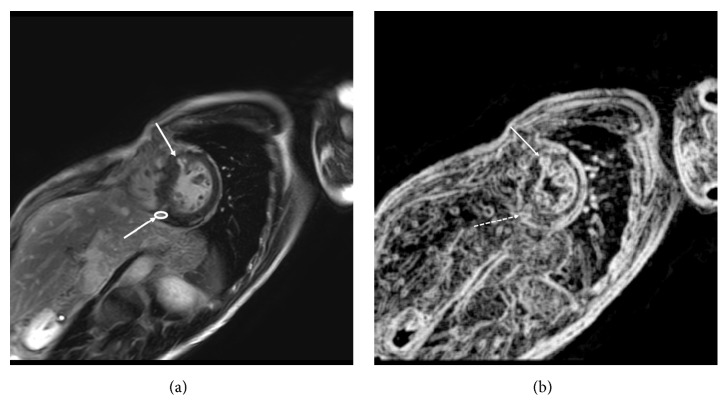
A 46-year-old man with hypertrophic cardiomyopathy and nonsustained ventricular tachycardia. (a) Five segments show late gadolinium enhancement (LGE; arrows). The white circle represents the region of interest for texture analysis. (b) The image of entropy LL is shown, and the entropy LL of LGE on this slice is 16557 (arrow) and 12771(dashed arrow), below the threshold of 19624.

The left ventricular myocardium was divided into 17 segments according to the American Heart Association (AHA) statement [19]. Therefore, a total of 391 myocardial segments were investigated in the 23 patients with HCM on LGE MR imaging. Myocardial LGE was visually identified by an investigator who was blinded to the presence or absence of ventricular tachyarrhythmia and had a 20-year experience in cardiac MR imaging and defined as a myocardium with a signal intensity 6SD greater than a remote normal-appearing myocardial signal. The same investigator also performed texture analysis of myocardial LGE using open-access MaZda software (version 4.5, Institute of Electronics, Technical University of Lodz, Poland) [10, 15, 17, 20]. A region of interest (ROI) with the 8 bits per pixel and 256 gray scales was placed into the area of the LGE segment where the enhancement was strongest to fit analyzed LGE and to avoid partial volume effects (Figure 1(a)) [15]. According to the previous studies [11, 14], we acquired 4 texture features out of approximately 100 features acquired using MaZda software including variance, skewness, kurtosis, and entropy LL of signal intensity of the myocardial LGE: variance, the change ratio of the pixel pair; skewness, distortion of a histogram; kurtosis, gathering condition to the average of histogram; and entropy LL, randomness in the low frequency. Another investigator, a referred cardiologist, determined the presence or absence of ventricular tachyarrhythmia.

### 2.4. Statistical Analysis

First, the difference in the numbers of LGE myocardial segments was assessed between the patients with and without the history of ventricular tachyarrhythmias, such as nonsustained ventricular tachycardia (NSVT) and ventricular fibrillation (VF). Second, we evaluated the differences in the 4 texture features between the patients with and without ventricular tachyarrhythmias. An unpaired* t*-test or Welch's* t*-test was used to evaluate the differences in the quantitative data between patients with and without ventricular tachyarrhythmias. The* p*-value less than 0.05 was defined as significant. Third, the threshold of the number of LGE segments or texture features to identify the history of tachyarrhythmias was assessed using a receiver-operating characteristic (ROC) analysis.

## 3. Results

Six (26.1%) of the 23 patients had a history of ventricular tachyarrhythmias: the one had a history of cardiac arrest because of VF and the other 5 patients showed NSVT. The remaining 17 of 23 patients did not have any histories of ventricular tachyarrhythmias. Eighty-two (21.0%) of the 391 myocardial segments showed LGE in 16 (69.6%) of the 23 patients, ranging from 0 to 13 myocardial segments per patient. For texture analysis, 43 ROIs were placed on the LGE segments because there were some LGE covering 2 or more segments contiguously and there were spotty LGE where we could not place ROI appropriately.

**Figure 2:**
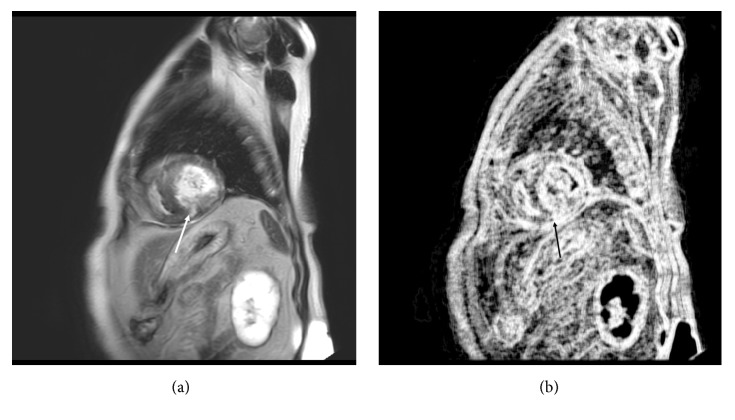
A 69-year-old man with hypertrophic cardiomyopathy (HCM) and a family history of HCM, but without ventricular tachyarrhythmias. (a) Three segments show late gadolinium enhancement (LGE; arrow). (b) The image of entropy LL is shown, and the entropy LL of LGE on this slice is 26065, above the threshold of 19624 (arrow).

**Figure 3:**
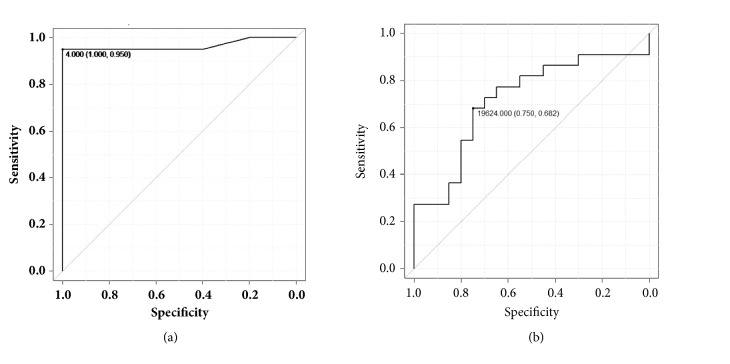
Receiver-operating characteristic analysis shows that 4 segments with late gadolinium enhancement (a) and an entropy LL of 19624 (b) are appropriate thresholds to discriminate between patients with and without a history of ventricular tachyarrhythmias. Parentheses show sensitivity and specificity.

Table 1 summarizes the number of LGE segments and 4 texture features in the 23 patients with HCM. The number of LGE segments was significantly greater in patients with ventricular tachyarrhythmias (8.2 ± 3.3) than in those without (1.9 ± 2.7;* p* = 0.0002). Six patients with HCM and ventricular tachyarrhythmias had 21 ROIs for the texture analysis, while the remaining 17 patients had 22 ROIs. Among the 4 texture features, entropy LL was significantly smaller in patients with the arrhythmias (14970 ± 9108) than in those without (25565 ± 14085;* p* = 0.0058). There were no significant differences in the other 3 texture features between the 2 patient groups. The ROC analysis showed that the 4 segments with LGE (area under the curve [AUC] = 0.96) and the entropy LL of 19624 (AUC = 0.72) were appropriate thresholds to discriminate the patients with and without a history of ventricular tachyarrhythmias (Figures 1–3).

## 4. Discussion

The present study demonstrated that the number of LGE segments and entropy LL of LGE differed between HCM patients with and without a history of ventricular tachyarrhythmias, such as NSVT and VF. In addition, we determined the thresholds of these factors to discriminate the 2 patient groups. Therefore, the extension of LGE as well as its texture features may be significantly related to the ventricular tachyarrhythmias in patients with HCM.

Myocardial LGE on cardiac MR imaging indicates myocardial fibrosis in patients with HCM [21]. Previous studies demonstrate that the presence or extension of LGE is related to ventricular tachyarrhythmia, indicating that the myocardial fibrosis in HCM is of an arrhythmic origin HCM [5–8]. The present study also showed that the number of LGE segments was significantly greater in HCM patients with arrhythmias than those without and that 4 segments of LGE were the threshold to discriminate patients with and without a history of ventricular tachyarrhythmias. Therefore, HCM patients with more than 4 segments showing LGE may be at risk of serious arrhythmia.

In this study, we also used texture analysis to identify LGE related to ventricular tachyarrhythmias. Among 4 texture features, only entropy LL was a discriminator between the 2 HCM patient groups with a threshold of 19624. Entropy LL was significantly less in patients with a history of ventricular tachyarrhythmias than in those without, indicating that LGE with microscopical homogeneity and less randomness was related to arrhythmia in HCM. Previous studies show that intermediate-signal-intensity LGE (i.e., gray zone) or inhomogeneous LGE, probably reflecting the mixture of fibrosis and normal myocardium, is significantly related to serious ventricular arrhythmia associated with ischemic cardiomyopathy and HCM [9, 22]. By contrast, Zhang et al. [23] have indicated that the clinical significance of gray zone and LGE burden may differ between ischemic and nonischemic cardiomyopathies. Fukushima et al. [24] have shown that scarred apical aneurysm can induce ventricular tachyarrhythmias in patients with HCM. Our study indicates that the wider extension of microscopically homogeneous fibrosis, probably replacement fibrosis, is related to ventricular tachyarrhythmias in patients with HCM. The advantage of texture analysis may be its ability to give more objective and quantitative data about the LGE than the SD threshold methods in HCM, where the remote nullified myocardium (i.e., normal-appearing myocardium) on LGE MR imaging can include interstitial fibrosis in this nonischemic cardiomyopathy. In addition, there is some difficulty defining “intermediate-signal-intensity” in HCM, as far as the definition of LGE is made by the SD threshold methods.

There were some limitations to the present study. First, the sample size of HCM was relatively small. Nonetheless, we were able to analyze 4 texture features of 43 ROIs on the 82 LGE areas in the HCM patients. Second, 2 types of magnetic field strength were used. Therefore, we assessed the extension of LGE according to the AHA segmentation model rather than the LGE mass, whereas the same 6SD threshold was used to identify LGE both on 1.5T and on 3.0 T. Nevertheless, the present study showed that the numbers of LGE segments and entropy LL were significantly related to ventricular tachyarrhythmias in patients with HCM. Third, we did not analyze all texture features acquired with the software, because the data handling may be too excessive (i.e., approximately 100 texture features) to be used clinically [14, 17]. Further study with a fast and powerful computer will be necessary to determine whether some of 100 texture features provide a higher AUC than the extension of LGE for identifying the patients with HCM and ventricular tachyarrhythmia. In addition, the threshold of entropy LL or texture features discriminating the HCM patients with and without ventricular tachyarrhythmias may differ from that of the present study if other software is used for texture analysis of LGE images [25]. Finally, we did not compare between the LGE showing low or high values of entropy LL and the histological findings. Myocardial LGE of HCM certainly reflects collagen or replacement fibrosis, but the presence of interstitial fibrosis and inflammatory process within LGE areas is controversial [21, 26, 27]. It remains unknown whether patchy myocardial fibrosis having compact groups of strands, which induces activation delay, is consistent with myocardial LGE with less entropy LL in patients with HCM and ventricular tachyarrhythmias [28].

In conclusion, HCM patients with a history of ventricular tachyarrhythmias had a wider extension of LGE, and the entropy LL of LGE was significantly less than in those without. The extension of LGE and texture analysis may provide information about LGE related to ventricular tachyarrhythmias in HCM. Based on the present ROC analysis, the extension of LGE may provide a better result than texture analysis in relation to the history of ventricular tachyarrhythmias associated with HCM.

## Figures and Tables

**Table 1:** Clinical and MR imaging features of 23 patients with hypertrophic cardiomyopathy.

	VTA (+)	VTA (-)	P value
no.	6	17	
sex	6 men	13 men, 4 women	NA
age	54.8 ± 13.1 (38-73)	64.2 ± 12.6 (46-90)	0.14
LGE segment no.*∗*	8.2 ± 3.3 (5-10)	1.9 ± 2.7 (0-11)	0.0002
Texture features			
variance	410.6 ± 417.8	403.8 ± 307.5	0.95
	(25.4-1431.6)	(72.7-1089)	
skewness	−0.19 ± 0.42	−0.41 ± 0.50	0.17
	(-0.87-0.65)	(-1.33-0.45)	
kurtosis	−0.47 ± 3.3	−0.17 ± 0.74	0.11
	(-1.44-0.46)	(-1.28-1.19)	
entropy LL*∗*	14969.9 ± 9107.6	25565.2 ± 14085.3	0.0058
	(5080-35745)	(2523-53379)	

VTA: ventricular tachyarrhythmias; no.: number; NA: not available; LGE: late gadolinium enhancement. Parentheses represent the range of values. *∗*Number of LGE segments and an entropy LL significantly differed between hypertrophic cardiomyopathy patients with and without a history of ventricular tachyarrhythmias.

## Data Availability

The data used to support the findings of this study are available from the corresponding author upon request.
